# High-Resolution Functional Mapping of the Venezuelan Equine Encephalitis Virus Genome by Insertional Mutagenesis and Massively Parallel Sequencing

**DOI:** 10.1371/journal.ppat.1001146

**Published:** 2010-10-14

**Authors:** Brett F. Beitzel, Russell R. Bakken, Jeffrey M. Smith, Connie S. Schmaljohn

**Affiliations:** The United States Army Medical Research Institute of Infectious Diseases, Fort Detrick, Maryland, United States of America; University of Texas Medical Branch, United States of America

## Abstract

We have developed a high-resolution genomic mapping technique that combines transposon-mediated insertional mutagenesis with either capillary electrophoresis or massively parallel sequencing to identify functionally important regions of the Venezuelan equine encephalitis virus (VEEV) genome. We initially used a capillary electrophoresis method to gain insight into the role of the VEEV nonstructural protein 3 (nsP3) in viral replication. We identified several regions in nsP3 that are intolerant to small (15 bp) insertions, and thus are presumably functionally important. We also identified nine separate regions in nsP3 that will tolerate small insertions at low temperatures (30°C), but not at higher temperatures (37°C, and 40°C). Because we found this method to be extremely effective at identifying temperature sensitive (ts) mutations, but limited by capillary electrophoresis capacity, we replaced the capillary electrophoresis with massively parallel sequencing and used the improved method to generate a functional map of the entire VEEV genome. We identified several hundred potential ts mutations throughout the genome and we validated several of the mutations in nsP2, nsP3, E3, E2, E1 and capsid using single-cycle growth curve experiments with virus generated through reverse genetics. We further demonstrated that two of the nsP3 ts mutants were attenuated for virulence in mice but could elicit protective immunity against challenge with wild-type VEEV. The recombinant ts mutants will be valuable tools for further studies of VEEV replication and virulence. Moreover, the method that we developed is applicable for generating such tools for any virus with a robust reverse genetics system.

## Author Summary

Venezuelan equine encephalitis virus (VEEV) is a New World *Alphavirus* that was first identified in Venezuela in 1938. VEEV normally circulates in rodent populations, but during outbreaks it can jump to horses and humans where it can cause debilitating and potentially fatal disease. There are currently no vaccines or antiviral agents against VEEV licensed for use in humans. In this study, we describe a technique that we have developed that allows for the rapid identification of viral mutants that can be useful for studying the basic biology of viral replication. These mutants can also be used to generate vaccines that protect against infection with wild-type virus. We demonstrate the utility of this technique by identifying over 200 mutations spread throughout VEEV genome that make the virus unable to replicate efficiently at higher temperatures (37°C or 40°C.) Furthermore, we show that two of the mutant viruses work as vaccines, and protect mice against lethal infection with VEEV. This technique can be applied to studying other viruses, and may allow for the rapid identification of numerous vaccine candidates.

## Introduction

Venezuelan equine encephalitis virus (VEEV) is a New World *Alphavirus* endemic to regions of South America. Normally maintained in a rodent reservoir, VEEV can be transmitted by mosquitoes to horses and humans where it can cause debilitating and potentially fatal encephalitis. There are currently no vaccines for VEE licensed for use in humans.

Alphaviruses contain an approximately 11–12 kb single-strand, capped and polyadenylated positive-sense RNA genome. The 5′ two-thirds of the genome encode the non-structural proteins; nsP1, nsP2, nsP3, and nsP4, which are involved in genome replication and transcription. The 3′ one-third of the genome encodes the structural proteins; capsid, E3, E2, 6K, and E1.

Much of what is known about the functions of *Alphavirus* non-structural proteins has been elucidated through molecular and classical genetics studies of two prototypical alphaviruses: Sindbis virus (SINV), and Semliki Forest virus (SFV) (reviewed in [1], [2], [3], [4]). The non-structural proteins are initially translated as two polyproteins, P123 and P1234. In SINV, and several other alphaviruses including VEEV, the major non-structural polyprotein, P123, is produced by translation termination at an opal codon at the end of nsP3. Occasional read-through of the opal termination codon produces P1234. Cleavage of P1234 in cis by a protease activity that resides in nsP2 generates a complex of P123 and nsP4 that can initiate minus-strand RNA synthesis. P123 is cleaved into nsP1, nsP2, and nsP3, and these fully cleaved forms generate a complex with cellular proteins, replicate the full-length viral genome from minus-strand templates, and transcribe the subgenomic RNA encoding the viral structural proteins.

The enzymatic activities and functional roles of nsP1, nsP2, and nsP4 have been partially characterized. nsP1 has methyltransferase and guanylyltransferase activity [5], [6], [7], [8], [9], required for capping RNA, and is necessary for synthesis of minus-strand RNA [10]. nsP2 has multiple functions in viral replication. It has RNA helicase activity [11] and NTPase activity [12], [13], and the C-terminus of nsP2 functions as a cysteine protease that cleaves the non-structural polyproteins P123 and P1234 [14], [15], [16], [17]. nsP2 has been found to enter cell nuclei and to be an inhibitor of transcription of cellular messenger and ribosomal RNAs including those involved in innate immune responses [18], [19], [20] nsP4 is the RNA dependent RNA polymerase [21], [22], [23], [24].

nsP3 is the least characterized of the *Alphavirus* nonstructural proteins. Studies with SINV showed that nsP3 is essential for both minus-strand and plus-strand synthesis, but the precise role that it plays in these activities is unknown [25], [26], [27]. The protein consists of two domains, an N-terminal macrodomain that is highly conserved among alphaviruses and a hypervariable, phosphorylated C-terminal domain. Macro domains (also called X domains) are found in proteins from bacteria, archea, and eukaryotes, as well as several viruses including alphaviruses, coronaviruses, hepatitis E virus, and rubella virus [28], [29], [30], [31]. They bind ADP-ribose and poly(ADP-ribose) and exhibit ADP-ribose 1″-phosphatase activity. The crystal structure of the VEEV macrodomain was recently solved, revealing a conserved adenosine binding pocket [32]. nsP3 is the only *Alphavirus* non-structural protein that is phosphorylated [33], with most or all of the phosphorylation on serine and threonine residues in the C-terminus [34], [35].

The studies that we report here were initiated to gain insight into the function of VEEV nsP3. Toward this goal, we used transposon mutagenesis, reverse genetics, and fragment analysis by capillary electrophoresis to identify regions of the nsP3 gene that are important for replication and that result in temperature sensitive (ts) mutations. Although this work demonstrated the utility of using insertional mutagenesis for identifying such regions, the method was limited by the inherently low capacity of fragment analysis by capillary electrophoresis. Consequently, we developed a novel high-resolution functional mapping technique that couples transposon insertional mutagenesis with tag sequencing on a next-generation sequencing platform. We used transposon mutagenesis to construct a cDNA library with small DNA fragments randomly inserted throughout the VEEV genome and then produced replication-competent virus through reverse genetics. Comparing transposon insertion sites in the resultant viruses to those in the starting library, we were able to produce a functional map of the entire genome of VEEV, and to identify several hundred potential ts mutations, including those we originally identified with the capillary electrophoresis method. We further validated the mutations in cell culture assays of recombinant viruses generated through reverse genetics and tested two of the nsP3 ts mutants in mice to see if they could act as attenuated vaccines. These studies provide both new information about the association of nsP3 gene regions with virulence, as well as a rapid and effective method for identifying and creating new ts mutants for replication studies.

## Results

### Construction and analysis of the nsP3 insertion library

**Figure 1:**
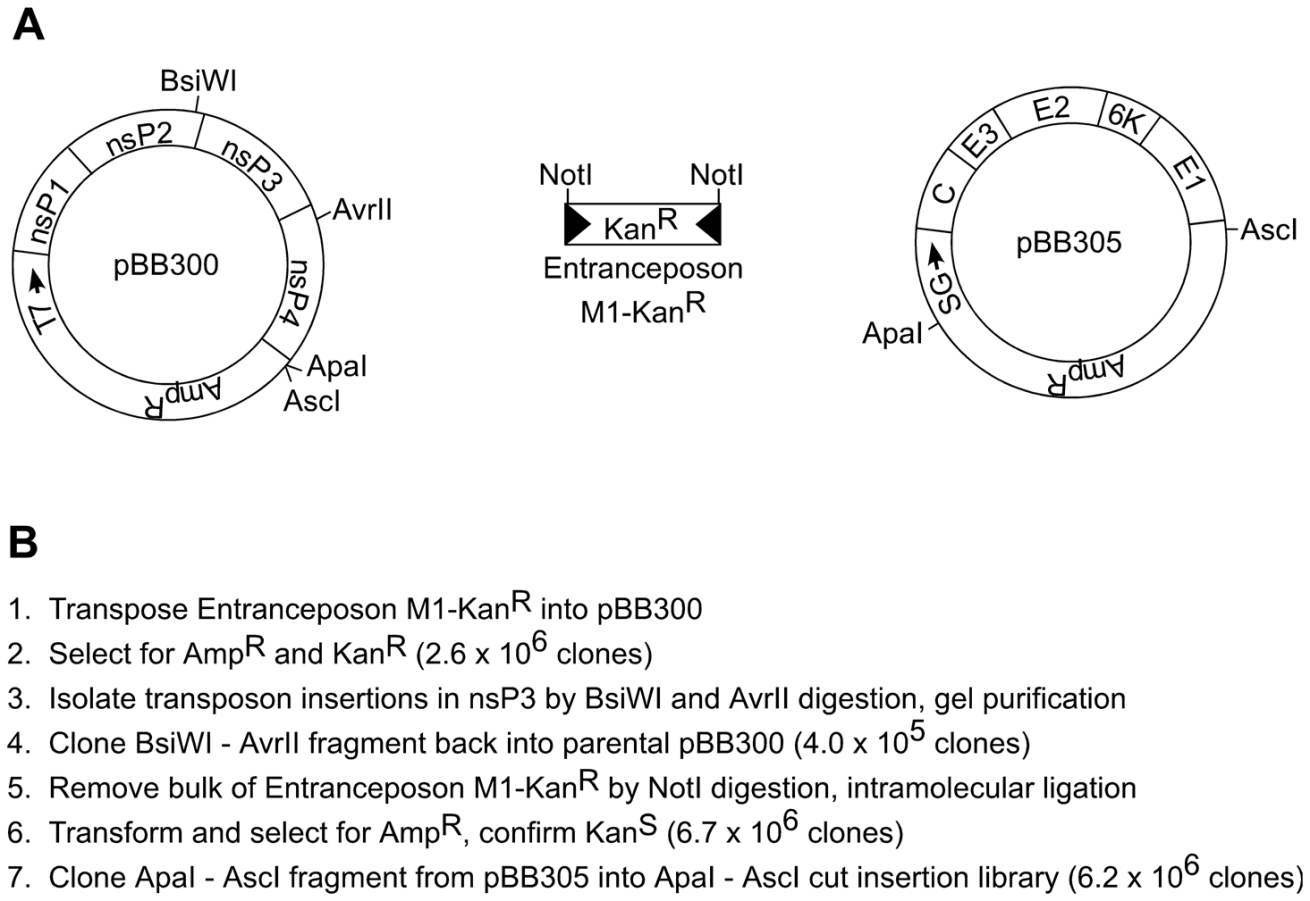
Generation of VEEV nsP3 insertion library. Entranceposon M1 – Kan^R^ was transposed into pBB300 and then processed to generate a library of clones that contained 15 base-pair inserts in nsP3 (and short flanking regions in nsP2 and nsP4). (A) Diagram of plasmids pBB300 and pBB305 and Entranceposon M1 – Kan^R^. T7 - T7 promoter; nsP1-nsP4 – VEEV non-structural proteins; SG – VEEV subgenomic promoter; C, E3, E2, 6K, E1 – VEEV structural proteins. (B) Procedure for making full-length VEEV library with short insertions in nsP3.

**Figure 2:**
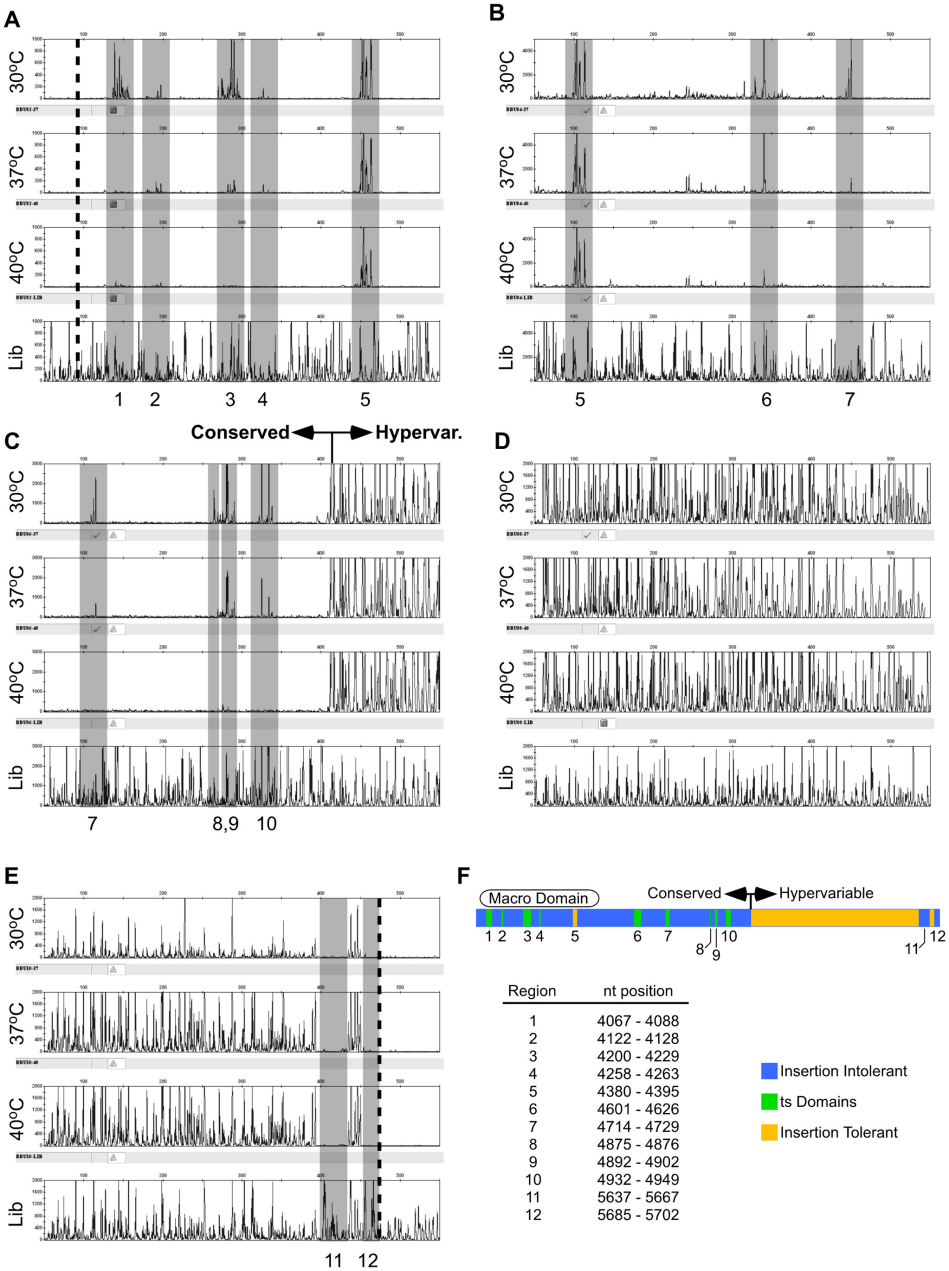
Functional mapping by capillary electrophoresis. Electropherogram data from five different amplicons spanning nsP3 are shown in panels A-E and summarized in panel F. The X axis is the DNA fragment size, and the Y-axis is relative fluorescence (proportional to the number of integrations at that site). From top to bottom in each panel, the electropherograms represent virus grown at 30°C, 37°C, and 40°C, and the unselected control (lib). Regions of interest are shaded in grey. The approximate beginning and end of nsP3 are indicated by dotted lines in panels (A) and (E), respectively. The end of the conserved region, and the beginning of the hypervariable region are indicated in panel (C). (A) Amplicon BBU02+BBU017, nts 3932–4684; (B) Amplicon BBU04+BBU018, nts 4281–5059; (C) Amplicon BBU06+BBU019, nts 4613–5350; (D) Amplicon BBU08+BBU020, nts 4961–5701; (E) Amplicon BBU010+BBU021, nts 5236–5803; (F) Compilation of nsP3 genetic mapping data. Regions intolerant to 15 bp insertions at all temperatures are shown in blue. Regions tolerant to insertions at 30°C, but intolerant at 37°C or 40°C are indicated in green. Regions tolerant to insertions at all temperatures are indicated in yellow. The locations of the macro domain, conserved region, and hypervariable region are shown.

We used a multistep transposon mutagenesis process to generate a library of full-length VEEV clones with single insertions in nsP3 (Figure 1). Our final library consisted of 6.2×10^6^ full-length clones of the VEEV genome with 15 bp insertions in nsP3 and had an average complexity of approximately 200-fold coverage at every nucleotide position. The 15 bp insertions consist of 10 bp derived from a modified MuA transposon and 5 bp derived from duplication of the transposition target site. The sequence derived from the transposon contains a unique *Not*I restriction site that allows for mapping the insert location in the VEEV genome. We produced virus pools from the insert library by using standard alphavirus reverse genetics, as depicted in Figure S1. Infectious RNA was produced by *in vitro* transcription of the insert library, and was transfected into BHK cells. The transfected BHK cells were then incubated at 30°C to generate virus particles. Virus was collected from the supernatant of the transfected cells and used to infect fresh Vero cells at an MOI of 0.1. The low MOI infection was intended to prevent trans-complementation caused by co-infecting mutant viruses, which could confound our downstream analyses. Infected cell cultures were incubated at either 30°C, 37°C, or 40°C to determine if there would be a temperature-dependent difference in the functional maps of viruses produced at these temperatures. RNA from our starting unselected pool, and RNAs isolated from the 30°C, 37°C, and 40°C infection supernatants were reverse transcribed and used as templates for PCR amplification using primer combinations (Tables S1 and S2) that generated amplicons of approximately 700 bp long with a fluorescent FAM label on the 5′ end, and a biotin tag on the 3′ end. The amplicons were bound to streptavidin-coated magnetic beads, and then digested with *Not*I to release any fragments containing a *Not*I site derived from a transposon insertion. The sizes of the released fragments were analyzed by capillary electrophoresis on a Prism 3130XL Genetic Analyzer. We included a FAM-labeled sequencing ladder of each amplicon to accurately size fragments. To analyze results, we generated electropherograms (Figure 2) in which the X-axis indicates the size of the DNA fragment (the transposon insertion position), and the Y-axis indicates the fluorescence intensity (the number of transposon insertions at that position).

Examination of the electropherograms from the starting, unselected library showed that it contained insertions after approximately 30–40% of the nucleotides in nsP3. The observation that less than half of the nucleotide positions in nsP3 had an insertion appeared to be due to a bias in MuA transposition, and not inadequate coverage in the library, as a second, independent insertion library gave a virtually identical insertion pattern (data not shown).

Comparison of the library electropherograms to those derived from viral genomes clearly revealed regions in nsP3 that would and would not tolerate 15 bp insertions. As expected, most regions of the highly conserved 5′ end of nsP3 would not tolerate insertions. Only one region in the 5′ end, from nucleotides 4380 to 4395 (relative to the genome of the Trinidad donkey strain of VEEV, Genbank accession number L01442), would tolerate insertions at all temperatures (region 5, Figures 2A and B).

In contrast, the hypervariable 3′ end of nsP3 tolerated insertions at most positions. From nt 5023 (near the start of the hypervariable region indicated in Figure 2C) to nt 5628, there was no discernable difference in the vRNA electropherograms compared to the unselected library RNA electropherogram. However, we did find two regions in the 3′ end, one from nucleotide positions 5637 to 5667 (region 11), and the other from 5685 to 5702 (region 12) that were intolerant of insertions. We were also able to detect 9 different sites in the 5′ end of the nsP3 gene that would tolerate insertions at 30°C, but not at 37°C or 40°C (summarized in Figure 2F). The degree of impairment of viral replication varied from site to site. Some of the sites allowed reduced replication at 37°C (i.e., detectable but reduced electropherogram peaks relative to the 30°C peaks), while others had no detectable replication at 37°C (e.g., compare Figure 2A regions 1 and 2). At 40°C, most sites had no detectable replication, but two had reduced replication (Figure 2B region 6 and Figure 2C region 9).

### Functional analysis of the entire VEEV genome

For whole-genome analysis by massively parallel tag sequencing, it was necessary to develop a modified sequencing library preparation protocol based on the Roche GS-FLX protocol such that the libraries had the sequencing adapter (adapter A) ligated onto the *Not* I sites that were present in the transposon insertions. A schematic of the protocol used for preparing the sequencing libraries is shown in Figure S2. Each prepared sequencing library (30°C virus, 40°C virus, and unselected *in vitro* transcribed RNA) was assessed on a single large region of a GS-FLX picotiter plate. We obtained 92,260 sequencing reads from the 30°C library, 276,722 reads from the 40°C library, and 161,936 reads from the unselected control RNA library. All analyses were normalized to account for the different number of sequences obtained from each sample. Sequencing reads arising from a transposon insertion should have a characteristic sequence tag at the 5′ end; thus, we analyzed the bulk sequences obtained for the presence of the correct tag, and those sequence reads lacking the tag were excluded from further analysis.

**Figure 3:**
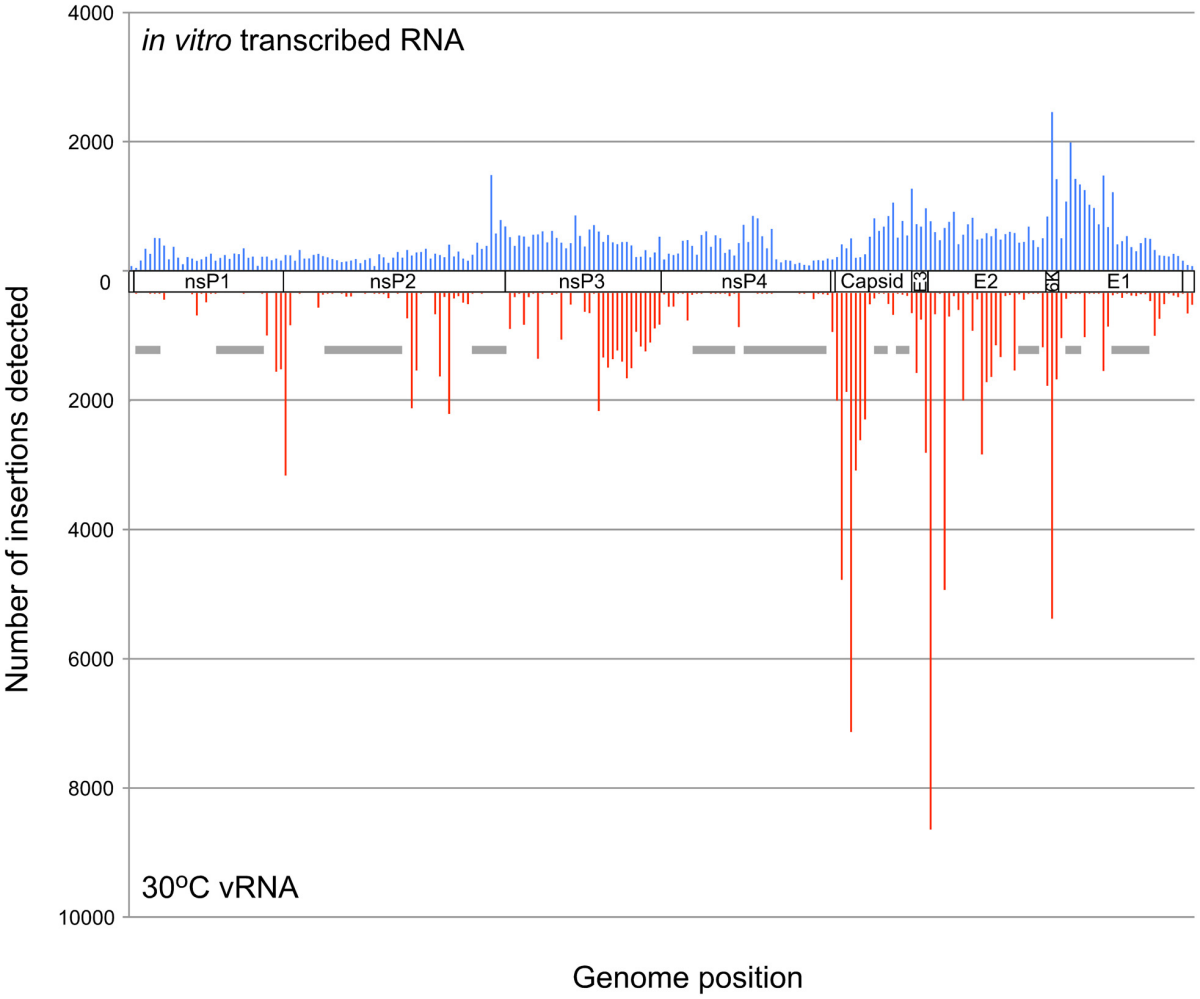
Frequency of insertion sites found in unselected RNA versus vRNA from virus produced at 30°C. The frequency of transposon insertions at each nucleotide position in the VEEV genome was calculated from the GS-FLX sequencing data and normalized to account for differences in the total number of sequencing reads obtained from each sample. For this histogram, the VEEV genome was divided into bins of 50 nucleotides from 5′ to 3′, and the total number of insertions in each bin was calculated. Insertion frequencies in unselected RNA are shown on top in blue, and vRNA isolated from virus produced at 30°C is shown in red. The approximate location in the genome is indicated between the two datasets. Gray bars indicate some of the regions intolerant to insertions at 30°C.

**Figure 4:**
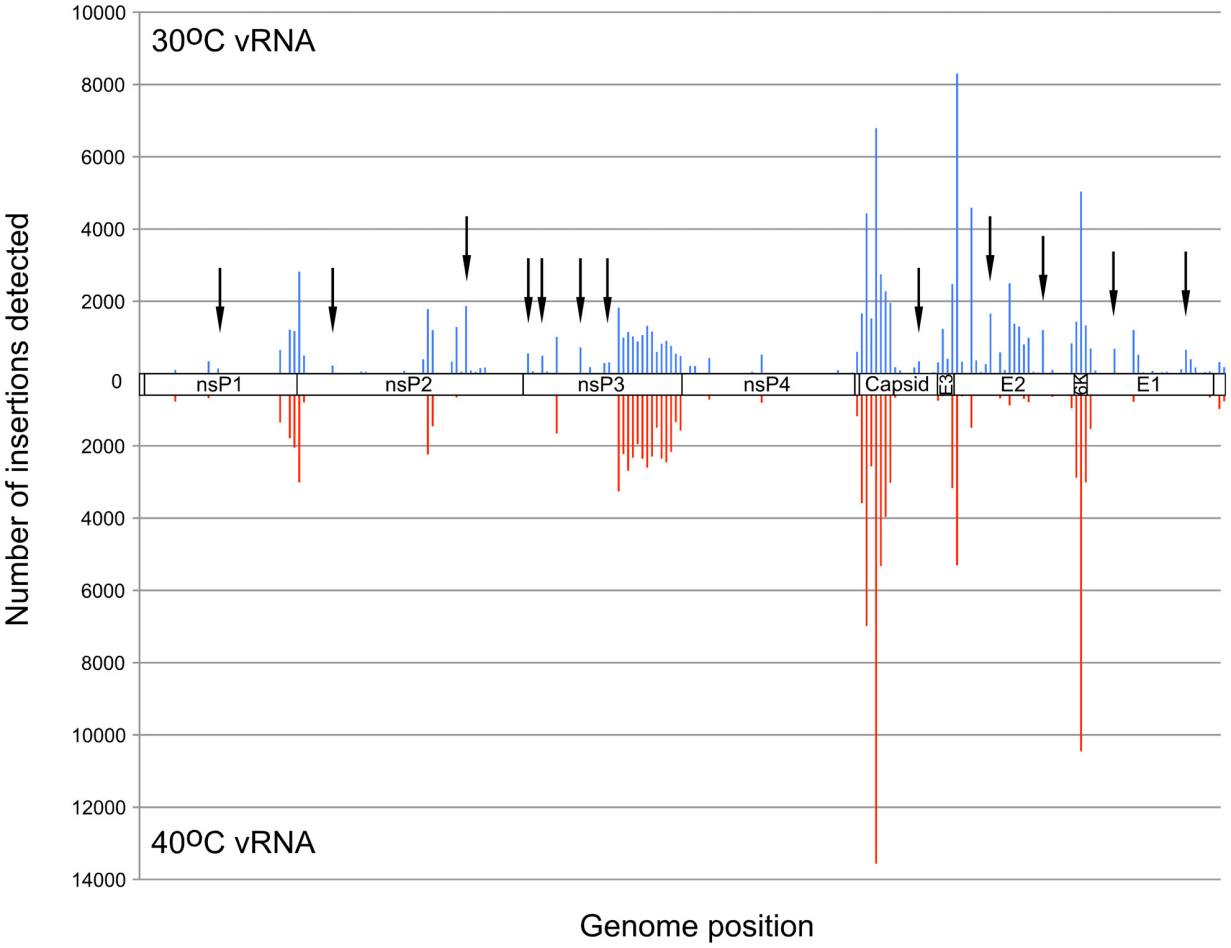
Frequency of insertion sites found in vRNAs of virus propagated at 30°C or 40°C. Transposon insertion frequencies were calculated for vRNAs isolated from 30°C and 40°C. Insertion frequencies for 50 nt bins are shown. 30°C frequencies are shown on top in blue, and 40°C frequencies are shown in red. Arrowheads indicate some of the regions in which more insertions were detected at 30°C than at 40°C.

**Figure 5:**
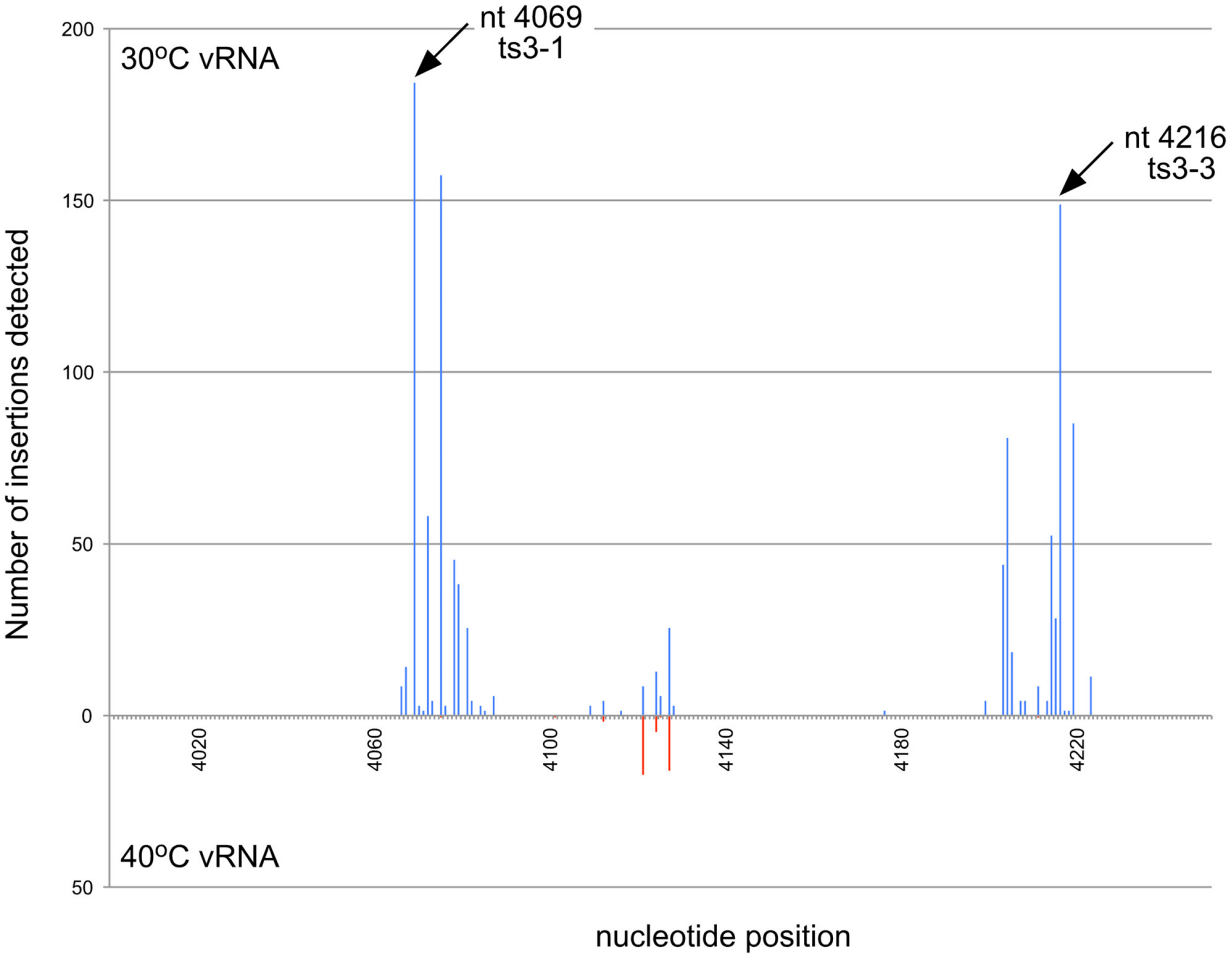
Location of nsP3 temperature sensitive mutants. The frequency of insertions at nt positions 4000–4250 at 30°C and 40°C is shown. The location of the ts mutants that were generated for the mouse challenge study are indicated with arrows. Mutants used in the study had either a single insertion at one of these locations (ts3-1 and ts3-3) or an insertion at both of these locations (double ts).

We used the first 20 nucleotides of each sequencing read to map the insert location on the VEEV genome. Because the VEEV genome is relatively small (∼11.5 kb), 20 bp was sufficient to uniquely identify the insert locations. The total number of insertions recorded at each nucleotide position in the genome was tallied and used to build a histogram of insertion frequencies across the entire genome (Data Set S1, and Figure 3 – 5).

As expected, the histogram of the unselected RNA sample showed insert locations spread throughout the entire genome (Figure 3). In contrast, the histogram of insert locations isolated from virus produced at 30°C showed several regions of the genome that did not tolerate insertions. These regions were presumably functionally important, and many of them mapped to domains with functions known to be required for viral replication, such as the nsP2 protease active site and substrate binding pocket [36], and the nsP3/4 protease cleavage site.

Although the histograms of the 30°C virus and the 40°C virus appeared quite similar, there were many locations within the genome where we detected insertions at 30°C, but not at 40°C (Figure 4), including those we identified in nsP3 using capillary electrophoresis analysis of the insertional library (Figure S3). In total, we found approximately 200 nucleotide positions within the genome where the ratio of insertions detected at 30°C was greater than or equal to 10 times the number of insertions detected at 40°C (Data Set S1).

### Construction of ts mutants

**Figure 6:**
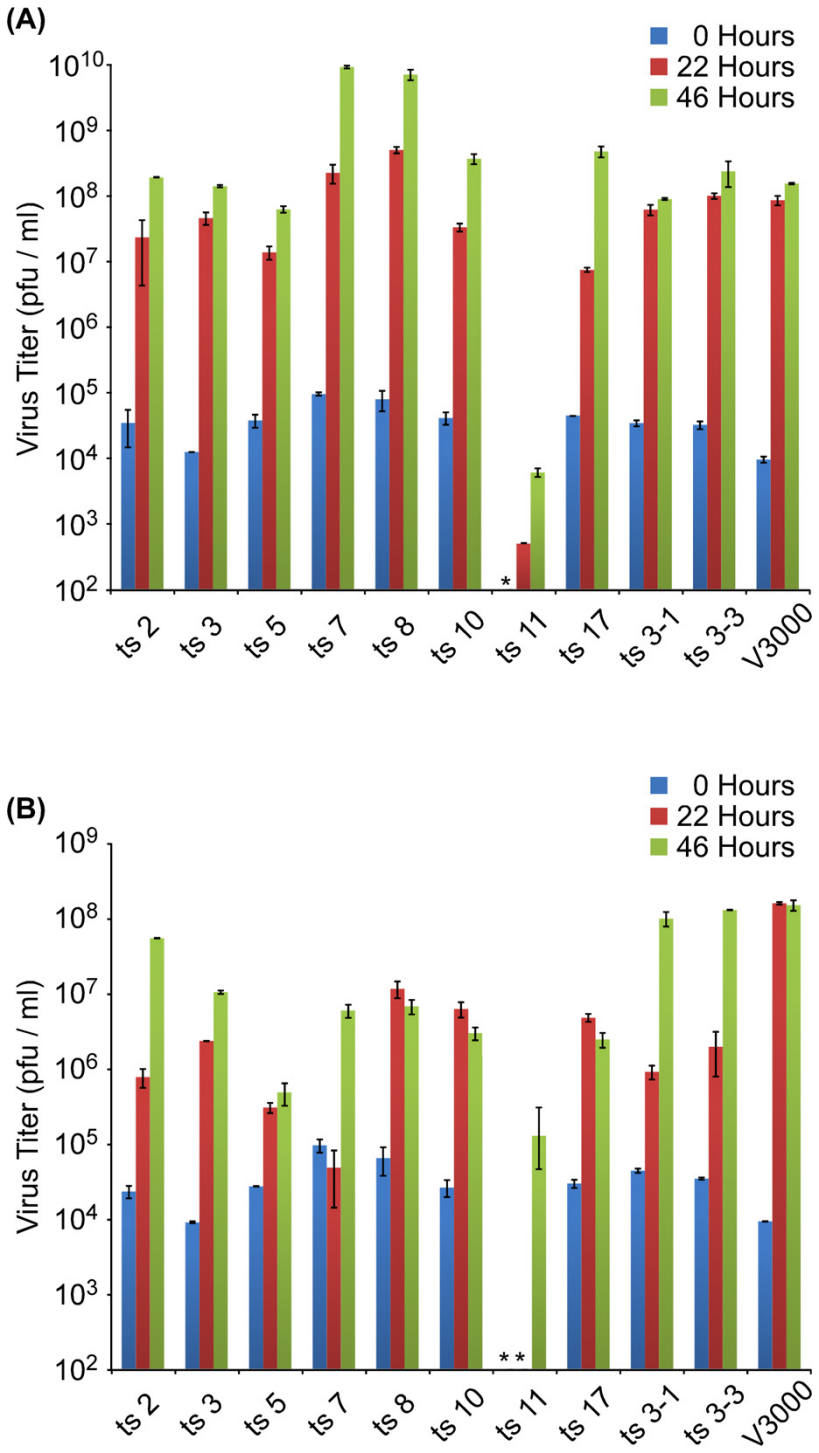
Virus replication at 30°C and 40°C. Single-cycle growth curves of viruses predicted to be ts based on functional mapping. Vero cells were infected in duplicate at an MOI of 1, and incubated at either 30°C (A) or 40°C (B). Aliquots were removed at 0, 22 and 46 hr after infection and virus was measured by plaque assay on Vero cells. Samples in which virus was not detected are indicated with an asterisk (*).

Using the data obtained by comparing the histograms of insert locations in the 30°C and 40°C vRNAs, we chose several locations in which insertions would be predicted to cause a ts phenotype. We constructed 10 mutants that contained 15 bp insertions that would mimic the insertions generated by a transposon insertion (Table 1.) We examined the single-cycle replication kinetics of each of these mutants at 30°C and 40°C, as compared to wild-type V3000. All of the mutants, with the exception of ts11, replicated at rates similar to wild type at 30°C (Figure 6). Two of the mutants, ts7 and ts8, replicated to higher titers than wild type at both 22 h and 46 h post infection. However, at 40°C, all of the mutants had titers that were reduced between 14- and 3300-fold relative to V3000 at 22 h post infection (excluding mutant ts11 which had undetectable titers at 22 h). At 46 h post infection, most of the mutants still had titers greater than 10-fold lower than wild-type V3000, but three mutants (ts2, ts3-1, ts3-3) had titers similar to wild type. Mutant ts11 had an extremely slow growth phenotype that made it difficult to measure. Plaques were barely visible at 4 days post infection, and took 7 to 10 days before accurate plaque counts could be obtained. Plaques from the other ts mutants were counted on day 3 post infection.

**Table 1:** Locations of temperature sensitive mutant insertions.

Mutant	Insert gene	Insert location	Insert	Amino Acid Sequence of Insert
ts2	nsP2	2969	TGCGGCCGCActggg	*_975_G-* CGRTG -*N_976_*
ts3	nsP2	3435	TGCGGCCGCAtccgc	*_1130_P-* LRPHP -*R_1131_*
ts5	E3	8432	TGCGGCCGCAcccat	*_290_P-* LRPHP -*C_291_*
ts7	E2	8921	TGCGGCCGCAcgtca	*_453_V-* MRPHV -*T_454_*
ts8	E2	9223	TGCGGCCGCAagtgc	*_554_C-* CGRKC -*T_555_*
ts10	E1	10474	TGCGGCCGCAatggg	*_971_G-* CGRNG -*V_972_*
ts11	E1	10905	TGCGGCCGCAtgcac	*_1115_T-* AAACT -*L_1116_*
ts17	Capsid	8153	TGCGGCCGCAccaag	*_197_Q-* VRPHQ -*G_198_*
ts3-1	nsP3	4069	TGCGGCCGCAgccac	*_1342_T-* AAAAT -*A_1343_*
ts3-3	nsP3	4216	TGCGGCCGCAgcagc	*_1391_A-* AAAAA -*K_1392_*

Genome locations of 15 bp inserts in temperature-sensitive mutants used in this study. The engineered insertions exactly mimic the expected insertions caused by transposon insertions at these locations. Bases arising from target site duplication are in lower case. The resulting amino acid insertions are indicated. Positions of flanking wild-type amino acids (in italics) are relative to either the non-structural or structural polyprotein.

**Table 2:** Serum neutralizing titers of groups inoculated with VEEV ts mutants.

Group	PRNT80*	Range
V3000, 10 PFU	226	<20 to >5120
ts3-1, 10 PFU	2658	<20 to >5120
ts3-1, 10000 PFU	4496	1920 to >5120
ts3-3, 10 PFU	2441	<20 to >5120
ts3-3, 10000 PFU	3795	2168 to >5120
Double ts, 10 PFU	3286	1197 to >5120
Double ts, 10000 PFU	2887	698 to >5120
PBS	<20	All <20

*80% plaque reduction neutralization titer, geometric mean.

### Infection of mice with ts mutant viruses

Temperature-sensitive mutants have been used extensively to elucidate replication and virulence properties of alphaviruses. To demonstrate the utility of our functional mapping method for identifying useful ts VEEV mutants, we further studied two nsP3 ts mutants, ts3-1 and ts3-3 for replication, virulence, and immunogenicity in mice. The insert locations in these two mutants are shown in Figure 5. In addition to these two mutants, we constructed a mutant with both the ts3-1 and ts3-3 insertions (double ts).

We infected groups of 10 Balb/c mice subcutaneously (s.c.) with either a low dose (10 pfu) or a high dose (10^4^ pfu) of ts3-1, ts3-3, double ts, or wild-type V3000. A negative control group was inoculated with PBS. All of the mice receiving the high-dose V3000 had to be euthanized when moribund on day 6, while 8 of 10 mice receiving the low-dose V3000 inoculation were euthanized when moribund by day 7. All of the mice that received either high doses or low doses of any of the three ts mutants survived without signs of disease, as did the negative control group.

As a verification that the ts mutants had actually infected the mice, we assayed their sera for the presence of neutralizing antibodies 28 days after infection. One mouse each in the low dose V3000, ts3-1, and ts3-3 groups had no detectable neutralizing antibodies, but all others did (Table 2 and Table S3).

**Figure 7:**
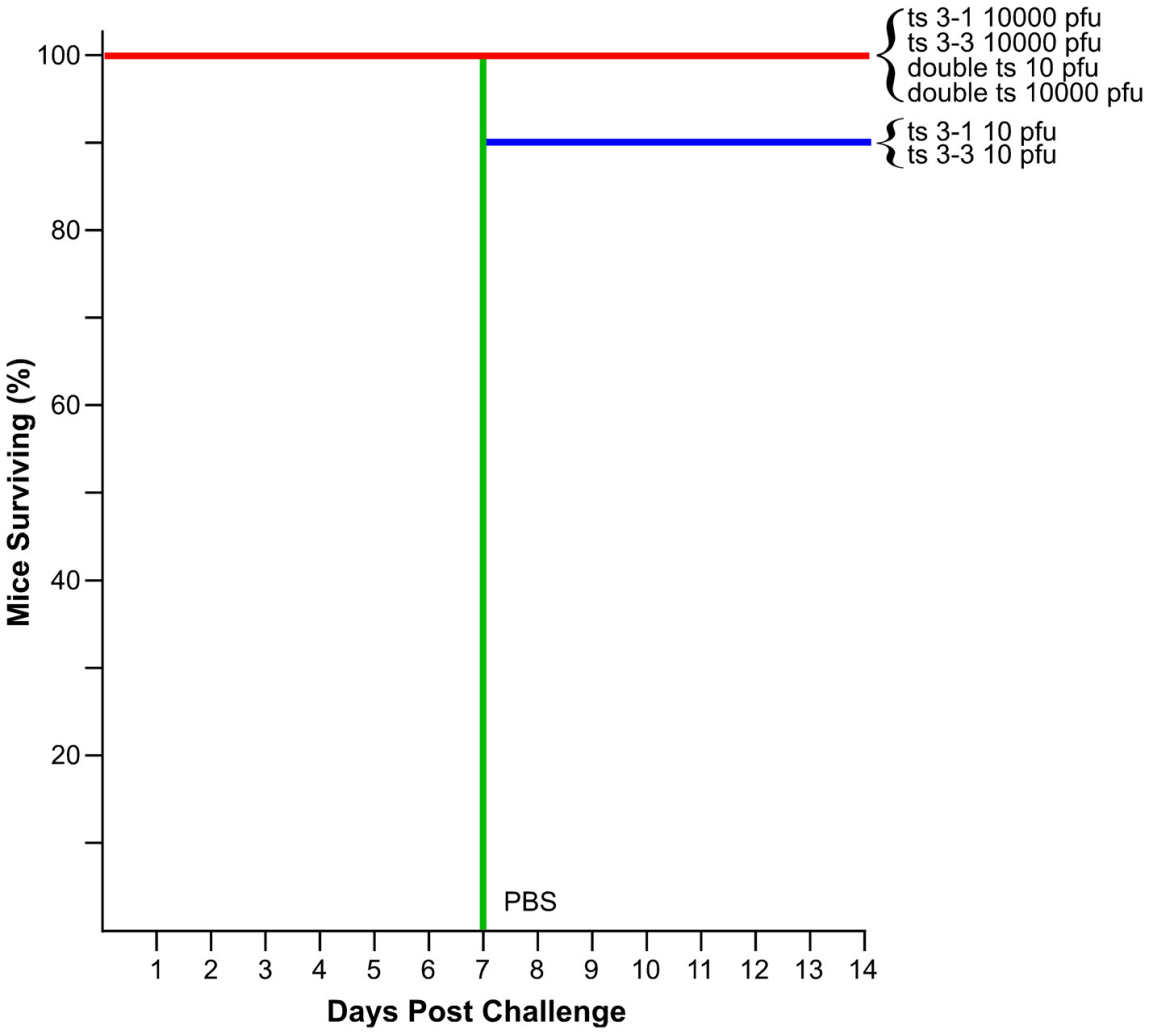
Kaplan-Meier analysis of mouse survival after challenge. Groups of 10 mice were innoculated with the VEEV ts strains and doses indicated along the right hand side of the graph, or with PBS. 28 days post innoculation, mice were challenged with 10^4^ pfu of wild-type VEEV strain Trinidad donkey and monitored for an additional 28 days. Mice were euthanized when moribund. All mice surviving to day 14 survived to day 28, when the study was terminated.

Neutralizing antibodies are a correlate of protective immunity to VEEV; thus we were interested in determining if the ts mutants were able to serve as protective, live-attenuated vaccines. Consequently, we challenged all of the surviving mice with 10^4^ pfu of VEEV, strain Trinidad donkey, 28 days after their initial infection with the ts mutants (Figure 7). All 10 of the negative control mice that had received PBS in the initial inoculation were euthanized when moribund on day 7 post challenge. One of the two mice that had survived the low-dose V3000 inoculation, as well as one mouse each in the low-dose ts3-1 and ts3-3 groups were euthanized when moribund on day 7 post challenge. All of the remaining mice survived for an additional 28 days post challenge without displaying disease symptoms.

## Discussion

The analysis of ts mutants has been used extensively to study *Alphavirus* replication, and has helped to identify the activities and interactions of many viral proteins [10], [21], [37], [38], [39], [40], [41], [42], [43], [44]. We originally planned to use functional mapping to gain insight into the role(s) that nsP3 plays during viral replication simply by mapping domains that would and would not tolerate short insertions. However, we found that by performing functional mapping on virus pools that had been produced at different temperatures, we could identify large numbers of ts mutants, which could be used for more in depth studies of viral replication.

Our mapping of nsP3 identified several interesting features. Not surprisingly, most of the conserved N-terminus of the protein was intolerant to short insertions while most of the variable C-terminus would tolerate insertions. Two regions in the C-terminus that would not tolerate insertions spanned nucleotides 5628–5666 and nucleotides 5684–5702. The latter region might be expected to be intolerant to insertions, as that region encodes the extreme C terminus of nsP3, including the cleavage site between nsP3 and nsP4. Disruptions in this region could prevent cleavage of nsP3 from nsP4, which is an essential step in viral replication [45]. The other site is more enigmatic. The region from 5628–5666 has no obvious homology to known regions of importance, and will require additional study to determine why insertions are not tolerated. One recent study with SFV indicated that the extreme C-terminus of nsP3 contains a degradation signal and a signal important for its' cellular localization [46]. The regions we have identified here might play a similar role in VEEV replication.

In addition to these two intolerant regions, we also identified nine separate regions in the 5′ end of nsP3 that are ts for insertions. Several of these regions mapped near ts regions identified in SINV [21], [25], [26], [47], but ts regions 1, 6, 9, and 10 were in regions that were not previously identified. Construction of these ts mutants through reverse genetics will be required to more fully characterize their roles in viral replication.

To generate a functional map of nsP3, we used fragment analysis by capillary electrophoresis to generate functional maps in a manner similar to one reported earlier [48], [49]. While this technique worked relatively well for analyzing short stretches of DNA or RNA (i.e., up to 2 kb), the technique had limitations that made it cumbersome for analysis of larger gene regions. For example, we could only analyze individual amplicons of approximately 600–800 bp due to the inherent loss of size resolution in larger fragments. We also needed to include a sequencing ladder in each electrophoretic analysis to obtain accurate fragment size information. Because our mapping fragments had short transposon-derived sequences appended to the 3′ end, fragment sizing with the sequencing ladder was not exact. We theorized that functional mapping by using massively parallel sequencing might overcome some of these issues. In principle, the large number of sequencing reads obtained would allow us to measure the relative frequencies of insertions at each nucleotide position in the genome. Massively parallel sequencing would also provide an exact sequence readout of the locations of inserts and generate highly accurate maps. Lastly, mapping by massively parallel sequencing would enable large stretches of DNA or RNA to be examined in a single sequencing run.

For our studies, we used a Roche GS-FLX system, which was adequate for mapping the approximately 11.5 kb VEEV genome (∼100 K–200 K reads per sample.) Viruses with larger genomes could be mapped by using other sequencing platforms such as the Illumina Genome Analyzer or Applied Biosystems SOLiD sequencer. These platforms produce hundreds of millions of sequencing reads, and would permit the analysis of a much larger number of samples per sequencing run. Therefore, they are probably the optimal systems for this type of analysis. Although the length of sequencing reads obtained on these systems are shorter than those obtained on the GS-FLX, the 35+ bp reads that they generate should be enough to map insertion sites onto the relatively simple genomes of viruses. In our analysis of VEEV, we found that the first 20 nts of sequence was sufficient for mapping each read back to the genome.

Functional mapping of the VEEV genome by using the massively parallel sequencing method that we developed revealed several hundred sites, spread throughout the genome in every gene, where the number of insertions detected at 30°C was greater than 10 times higher than the number of sequences detected at 40°C. All 10 sites that we chose for further analysis gave rise to a virus with a ts phenotype, thus it is likely that most or all of the other sites could also be reverse engineered to generate novel ts mutant viruses that could be used for functional studies of VEEV proteins. Many of the sites fell outside of the known functional domains of VEEV proteins, such as the helicase and peptidase domains of nsP2 and the RNA-dependent RNA polymerase domain of nsP4, and may represent additional domains with unknown functions in these proteins. Elucidating the mechanisms of attenuation for these mutants will be important for identifying additional functions required for viral replication.

In addition to providing new ts mutants for studying viral replication, this method can also be used to map virulence properties. For example, we demonstrated one use of functional mapping data by constructing ts mutants that acted as attenuated vaccines in a mouse model of lethal VEEV infection. None of the mice vaccinated with these viruses showed any signs of disease, and most were protected from challenge with wild-type VEEV. Of the three mice that did succumb to challenge, all were in groups that were inoculated with only 10 pfu of ts mutant virus; thus, it is possible that these mice were not infected in the first place. This is supported by the observation that one mouse in each group did not develop neutralizing antibodies after infection with the ts mutants. The utility of this technique for designing ts vaccines lies not only in the ability to detect large numbers of ts mutants, but also in the ability to multiplex those mutations to generate viruses with a potentially more stable ts phenotype. In our study, we demonstrated that we could combine two mutants in our double ts mutant and still recover viable ts virus. It might be possible to combine more than two mutations, with each additional mutation reducing the reversion potential of the final attenuated virus.

Finally, although we only presented functional mapping data relating to ts phenotypes, functional mapping of other phenotypes is also possible using this technique. For example, comparison of functional maps generated from RNA isolated from infected cells to vRNAs isolated from the supernatant of infected cells might identify mutants defective for particle formation. As an additional example, we have performed preliminary functional mapping of VEEV propagated in mosquito cells (C6/36) to identify insertions that confer a species-specific replication phenotype, and have identified several insertions in the VEEV genome that appear to allow virus to replicate in Vero cells but not in C6/36 cells, and vice versa. Similar studies could be performed to examine animal-, organ-, or cell-specific replication characteristics of viruses. This method should provide a powerful and new means to generate tools for studying a myriad of characteristics of any virus with a robust reverse genetics system.

## Materials and Methods

### Viruses and cell cultures

BHK and Vero cells were cultured in Eagle's minimal essential medium (EMEM) supplemented with 10% fetal bovine serum (FBS). VEEV strain V3000 is derived from a molecular clone of the wild-type Trinidad donkey strain of VEEV.

### Generation of insertion libraries

For studies using only the nsP3 gene we used a VEEV reverse genetics system derived from plasmid pVEE Replicon 1.0 [50], which contains a T7 RNA promoter driving expression of the VEEV 5′ UTR and nsP1 through nsP4. pV3000 26S contains the 3′ end of the VEEV genome, including the subgenomic promoter, the structural proteins and 3′ UTR. To prevent interference with restriction digestions required for genetic analysis, *Not*I sites in both pVEE Replicon 1.0 and pV3000 26S were changed to *Asc*I sites by digestion with *Not*I followed by ligation of a linker with the sequence 5′-GGCCGGCGCGCC-3′. This change did not interfere with virus production from the full-length genome. The modified pVEE Replicon 1.0 was named pBB300, and the modified pV3000 26S was named pBB305.

A MuA transposon, Entranceposon M1-Kan^R^ (1131 bp, part of the Mutation Generation Kit from Finnzymes, Espoo, Finland), was transposed into pBB300. Briefly, target DNA, Entranceposon M1-Kan^R^, and MuA transposase were combined in transposition buffer, and the transposition was allowed to proceed for 1 h at 30°C. The transposase was then inactivated by heating to 75°C for 10 min. The transposition reaction was desalted on a Sephadex G-50 spin column and transformed into DH5α *E. coli*. A primary insertion library with full-length transposon insertions in pBB300 was isolated by plating the transformed cells on LB containing ampicillin and kanamycin. The Mutation Generation Kit has been optimized so that greater than 99% of the clones in the library will contain a single transposon insertion. A fragment containing transposon insertions in nsP3 (and small fragments of nsP2 and nsP4) was isolated from the primary insertion library by digestion with *Avr*II and *Bsi*WI. This fragment was cloned into *Avr*II-*Bsi*WI cut pBB300, yielding a secondary library that only contained full-length transposon insertions in nsP3. Digestion of the secondary library with *Not*I, followed by intramolecular ligation, generated a tertiary library consisting of clones with 15 bp inserts spread throughout nsP3. Finally, the 26S *Apa*I-*Asc*I fragment from pBB305 was cloned into the *ApaI-AscI* cut tertiary library, generating the final library of full-length VEEV genomes with 15 bp insertions in nsP3.

For studies using the entire VEEV genome, we constructed a plasmid containing cDNA representing the full-length genome of VEEV, strain Trinidad donkey (Genbank accession number L01442), named pBB306. pBB306 was generated by cloning the *ApaI-AscI* fragment from pBB305 (containing the VEEV structural proteins) into *ApaI-AscI* cut pBB300. pBB306 has a T7 promoter immediately upstream, and a unique *Asc*I site immediately downstream of the VEEV genome. After linearization of the plasmid with *Asc*I, infectious viral RNAs (vRNA) were produced by *in vitro* transcription with T7 RNA polymerase. Massively parallel sequencing identified 10 nucleotide differences from the published Trinidad donkey (accession #L01442) sequence as follows: nucleotide (nt) 4151 A to G, nt 6044 T to C, nt 7208 T to C, nt 9073 G to A, nt 9279 A to T, nt 9397 T to C, nt 9487 C to T, nt 9531 A to G, nt 11386 C to T, and deletion of nt 11409 T in the 3′ UTR.

Entranceposon M1-Kan^R^ was transposed into pBB306 and a primary insertion library generated as described above. A secondary library was prepared by digesting the primary library with *Asc*I and *Sbf*I to release the VEEV genome from the plasmid backbone. Size fractionation of this digest on an agarose gel allowed isolation of VEEV genomes containing transposon insertions, and removal of wild-type genomes lacking transposon insertions. The transposon-containing genomes were re-cloned into pBB306. This secondary library was digested with *Not*I, followed by intramolecular ligation, to produce a tertiary library consisting of clones with 15 bp inserts spread throughout the VEEV genome. At least 60-fold coverage of the VEEV genome was maintained in the library throughout the cloning process.

### Recombinant virus production

Recombinant VEEV was produced essentially as described previously [50], [51]. Briefly, the nsP3 only or full genome insertion libraries were linearized by *AscI* digestion, and transcribed *in vitro* using T7 RNA polymerase (Ribomax, Promega, Madison, WI). This RNA was introduced into BHK cells by electroporation and the cells were then propagated in EMEM +10% FBS at 30°C until noticeable cytopathic effects (CPE) were seen (approximately 2 days). Cell culture supernatant was centrifuged to remove cellular debris, aliquoted, and frozen. The titer of the BHK-produced virus was determined by plaque assay on Vero cells. In a second round of infection, Vero cells were infected at a multiplicity of infection (MOI) of 0.1 (low MOI) with BHK-produced virus. Cells were incubated at 30°C, 37°C or 40°C (for the nsP3 library), or 30°C or 40°C (for the full genome library) and supernatant was collected after extensive CPE was observed.

### RNA isolation and RT-PCR

Virus from 50 ml of supernatant from the low MOI infections was concentrated by ultracentrifugation. vRNA was isolated from the viral pellets by Trizol-LS extraction (Invitrogen, Calsbad, CA). *In vitro*-transcribed RNA that had not been used to produce virus was used as the unselected pool, and was processed in parallel with vRNAs. For capillary electrophoresis fragment analysis, RNAs were reverse transcribed with random hexamer primers, and the resulting cDNA was amplified by PCR in 100 µl reactions (Platinum PCR Supermix High Fidelity, Invitrogen) using the following combinations of primers (Table S2): BBU002 + BBU017, BBU004 + BBU018, BBU006 + BBU019, BBU008 + BBU020, BBU010 + BBU021. In all cases, the forward primer was labeled with 6-FAM to facilitate detection and the reverse primer was labeled with biotin for subsequent purification steps. The amplicons were designed to overlap so that regions that would be obscured near the ends of one amplicon could be analyzed in the overlapping amplicons.

For massively parallel tag sequencing, RNA was processed with the FirstChoice RLM-RACE kit (Applied Biosystems/Ambion, Austin, TX) to add a 5′-RACE adapter to the vRNAs, and reverse transcription was performed using a mixture of VEEV-specific primers and a 3′-RACE adapter. The resulting cDNAs were amplified by PCR, generating six overlapping amplicons (Table S2). The amplicons spanned the entire genome, and were designed to overlap so that primer binding sites that would be obscured at the ends of each amplicon could be analyzed in the overlapping amplicons. The primers used for PCR added *Asc*I restriction sites to both ends of each amplicon to facilitate sequencing library preparation.

### Capillary electrophoresis

Products from the RT-PCR reactions were processed with a PCR purification kit (Edge Biosystems, Gaithersburg, MD), to remove remaining primers, enzymes, and free nucleotides. Samples were resuspended in restriction endonuclease buffer (NEB, Beverly, MA) and bound to streptavidin-coated magnetic beads (Invitrogen) for 1 h at 37°C. After binding, beads were washed three times with 500 µl of restriction endonuclease buffer, and were resuspended in 50 µl of restriction buffer containing 5 units of *Not*I restriction enzyme (NEB). Digests were incubated at 37°C for 1 h. After digestion, bead eluates were desalted on a sepharose filtration spin column and dried. Dried samples were resuspended in 10 µl of deionized formamide with a ROX-labeled size standard (Geneflo 625, Chimerx, Milwaukee, WI). Samples were denatured at 95°C for 2 min, and then loaded onto a Prism 3130XL Genetic Analyzer (Applied Biosystems, Foster City, CA) for capillary electrophoresis. FAM-labeled dideoxy sequencing ladders of each amplicon were run in parallel to allow for accurate fragment sizing. Settings for the run were the same as the default settings for fragment analysis on a 50 cm POP-7 array, except that the run time was changed from 1800 s to 2200 s. Data from the capillary electrophoresis runs were analyzed with Genemapper software version 3.7 (Applied Biosystems) using the Local Southern setting for peak sizing.

### Library preparation for massively parallel sequencing

Amplicons that had been generated by RT-PCR were mixed in an equimolar ratio to a final mass of 10 µg. The mixture was digested with *Asc*I to generate ligatable ends on each amplicon. The *Asc*I was heat inactivated, and the digest mixture was purified over Sepharose to remove the small end fragments generated by *Asc*I digestion. The purified amplicons were ethanol precipitated and resuspended in a 5 µl ligation mix including T4 DNA ligase and T4 polynucleotide kinase, and the ligation was incubated overnight at 16°C. The product of this ligation was a collection of high molecular weight DNAs (hmwDNA) with sizes greater than approximately 15 kb as visualized on an ethidium bromide stained agarose gel. The hmwDNAs were processed with a GS-FLX library preparation kit (Roche) with a few modifications to the kit protocol. As per the protocol, hmwDNAs were nebulized to generate small fragments of double stranded DNA approximately 400–800 bp in size. The ends of these fragments were polished with T4 DNA polymerase and T4 polynucleotide kinase to produce blunt, phosphorylated ends. In a departure from the kit protocol, sequencing adapter “B” was ligated to both ends of the DNA fragments. The fragments were then digested with *Not* I (which cuts in the transposon insertions), and a modified sequencing adapter “A” was ligated onto the exposed *Not I* ends. At this point, we returned to the library preparation kit protocol to finish preparing the sequencing libraries for each sample. The final product for each was approximately 10 ng of sequencing library. Sequencing out from the “A” adapter present on each DNA fragment allowed us to identify the locations of the transposon insertions in each sample.

### GS-FLX sequencing and sequence analysis

The sequencing libraries were amplified onto sequencing beads using the Roche emPCR kit II. This kit produces templates for sequencing reactions starting from the “A” adapters. After cleanup of the emPCR reactions, sequencing beads were loaded onto a Picotiter plate and sequenced on the GS-FLX. Sequences from one large region of a Picotiter plate were obtained for each sample. The sequences obtained (between 92,000 and 276,000 per sample) were processed by a short PERL script to identify those that contained a sequence tag indicative of a “true” sequence from a transposon insertion event. The sequence of these tags was A(R)*CGGCCGCA. The first A was from the modified “A” sequencing adapter. The (R)* came from a multiplex identifier (MID) that was included at the 3′ end of the modified “A” adapter. The MID consisted of either four A or four G residues. The CGGCCGC was the residual *Not* I site, and the final A came from the 3′ end of the transposon insertion. Approximately 70% of the sequences from each sample contained this sequence tag. The sequence of the first 20 bases after the tag was BLASTed to the VEEV genome to identify the location of the insertion [52]. To be included in the final analysis, each BLAST result had to match the VEEV genome 100% over the 20 base sequence. Approximately 95% of the tag-containing sequences were successfully mapped to the VEEV genome using these criteria. The insertion sites identified by BLAST were tallied to build a histogram of insertion frequencies across the VEEV genome.

### Construction of temperature sensitive mutants

VEEV genomes carrying 15 bp insertions that mimicked a transposon insertion were constructed by using a Quikchange XL kit (Stratagene, La Jolla, CA.) Mutations were first constructed in plasmids containing subgenomic fragments. The mutations were confirmed by sequencing, and then transferred into a full-length genomic plasmid (pBB306) by restriction digestion and cloning. Mutant viruses were prepared from these full-length clones as described above.

### Growth curves

Vero cells plated in 6-well dishes were infected in duplicate with wild-type and mutant viruses at an MOI of 1. After binding the virus for 1 h at the appropriate temperature (30°C or 40°C,) the wells were washed once with PBS, and fresh medium was added. A sample was taken immediately after adding the fresh medium, and represented the 0 h time point. Infected cells were then cultured at either 30°C or 40°C, and additional samples were withdrawn from the supernatant at 22 h and 46 h post infection. All samples were frozen at −80°C prior to plaque assay.

### Vaccination and challenge of mice

Groups of 10 6-to 8-week-old Balb/c mice were inoculated s.c. with either 10 pfu or 10^4^ pfu of ts mutant viruses. Control groups received 10 or 10^4^ pfu of V3000 or PBS. Mice were weighed and examined daily for signs of disease, and euthanized when moribund. Twenty-eight days after the initial inoculation with the VEEV ts mutants, surviving mice were challenged s.c. with 10^4^ pfu of VEEV, strain Trinidad donkey. Mice were weighed and examined daily for signs of disease, and euthanized when moribund.

### Virus neutralization assays

Sera from vaccinated mice were serially diluted in Hank's Balanced Salt Solution (HBSS) containing 2% FBS. Serum dilutions were incubated overnight at 4°C with 200 pfu of VEEV Trinidad donkey. After incubation, the mixtures were assayed for plaque formation on Vero cells. The 80% plaque reduction neutralization titer (PRNT80) was calculated as the serum dilution at which plaque formation was reduced by 80% relative to a control that was incubated in the absence of serum.

### Ethics statement

The mouse research protocol was approved by the US Army Medical Research Institute of Infectious Disease Institutional Animal Care and Use Committee in compliance with the Animal Welfare Act and other federal statutes and regulations relating to animals and experiments involving animals and adheres to principles stated in The Guide for the Care and Use of Laboratory Animals, National Research Council, 1996. The facility where the research was conducted is fully accredited by the Association for Assessment and Accreditation of Laboratory Animal Care, International.

## Supporting Information

Figure S1 Transposon mutagenesis and alphavirus reverse genetics. A graphical representation of the protocol described in the text. A plasmid containing the entire VEEV genome (pBB306) was subjected to insertional mutagenesis by transposition of a modified MuA transposon (Entranceposon M1-Kan^R^ shown in the inset.) Removal of the bulk of the transposon by NotI digestion followed by intramolecular ligation leaves a library of clones, each containing a 15 bp insertion at an essentially random location in the genome (blue Xs shown in the 15 bp insert library). Each insertion contains a unique NotI site that can be used to map the insert location. The library is transcribed *in vitro* to produce infectious virus-like RNAs. These RNAs are transfected into cells, yielding recombinant viruses after 24–48 hours.
(0.09 MB PDF)

Figure S2 Processing of vRNAs for massively parallel sequencing. A graphical representation of the protocol described in the text. Viral genomic RNAs (vRNAs) are isolated from the supernatant of infected cell cultures. RACE and RT-PCR are used to generate amplicons that span the entire viral genome. The amplicons are mixed in an equimolar ratio, and digested with AscI (added by the primer during PCR) to generate 5′ phosphorylated ends. This mix is then ligated into a random jumble of high molecular weight DNA (hmwDNA) to generate a starting material for sequencing library preparation. The hmwDNA is nebulized into fragments 400–800 bp in size, and the ends are polished to generate blunt, 5′ phosphorylated ends. Sequencing adapter “B” (shown in green) from the Roche library preparation kit is ligated onto both ends of the polished DNAs. This mixture is then digested with NotI to expose the ends of the transposon insertions, and a biotinylated modified adapter “A” (shown in blue) is ligated onto the exposed NotI ends. The mix is bound to streptavidin coated magnetic beads and washed to remove any fragments lacking the biotin tag. Templates for GS-FLX sequencing are eluted from the magnetic beads as single stranded DNA.
(0.05 MB PDF)

Figure S3 Comparison of electropherogram data to results obtained by sequencing. Electropherogram data (top), and sequencing histogram (bottom) from approximately nt 4670 - nt 5220 in nsP3, showing that similar results are obtained using both techniques.
(0.10 MB PDF)

Table S1 Oligonucleotides used in this study.
(0.03 MB PDF)

Table S2 Amplicons used in this study.
(0.03 MB PDF)

Table S3 Serum neutralizing titers for individual mice vaccinated with VEEV ts strains. 80% plaque reduction neutralization titers (PRNT80) of individual animals after inoculation with VEEV ts mutants. Titers registered as <20 indicate that no neutralization activity was seen at the lowest serum dilution (1∶20). Titers registered as >5120 indicate that plaque numbers were reduced >80% (compared to a no-serum control) at the highest dilution used in the assay (1∶5120).
(0.03 MB PDF)

Data Set S1 Compiled sequencing data. Sequence reads obtained from GS-FLX sequencing were processed as described in the text. Columns F-H indicate the raw number of sequence reads detected at each nucleotide position in the VEEV genome. Columns I-K show the number of insertions per nucleotide position normalized to 100000 reads per sample.
(2.36 MB XLS)
