# Supplementary material for: High-Resolution Functional Mapping of the Venezuelan Equine Encephalitis Virus Genome by Insertional Mutagenesis and Massively Parallel Sequencing
Source: PLoS Pathog. 2010 Oct 14;6(10):e1001146. doi: 10.1371/journal.ppat.1001146 (PMC2954836; doi:10.1371/journal.ppat.1001146)
Supplement: Figure S1 — Transposon mutagenesis and alphavirus reverse genetics. A graphical representation of the protocol described in the text. A plasmid containing the entire VEEV genome (pBB306) was subjected to insertional mutagenesis by transposition of a modified MuA transposon (Entranceposon M1-KanR shown in the inset.) Removal of the bulk of the transposon by NotI digestion followed by intramolecular ligation leaves a library of clones, each containing a 15 bp insertion at an essentially random location in the genome (blue Xs shown in the 15 bp insert library). Each insertion contains a unique NotI site that can be used to map the insert location. The library is transcribed in vitro to produce infectious virus-like RNAs. These RNAs are transfected into cells, yielding recombinant viruses after 24–48 hours. (0.09 MB PDF) [file ppat.1001146.s001.pdf]

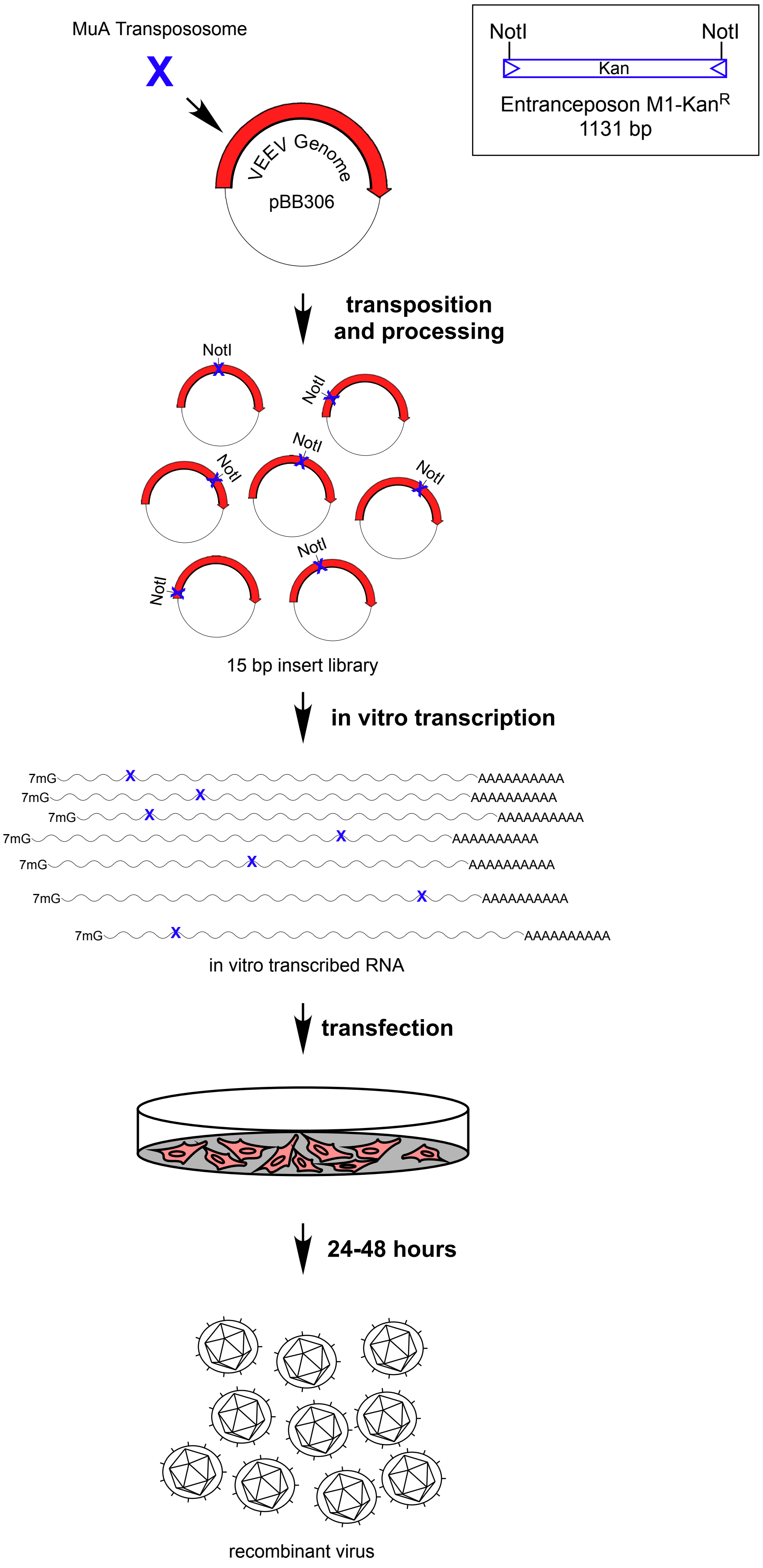
MuA Transpososome
NotI
NotI
Kan
Entranceposon M1-KanR
1131 bp
VEEV Genome
pBB306
transposition
and processing
NotI
15 bp insert library
in vitro transcription
7mG
AAAAAAAAAA
in vitro transcribed RNA
transfection
24-48 hours
recombinant virus
