# Supplementary material for: High-Resolution Functional Mapping of the Venezuelan Equine Encephalitis Virus Genome by Insertional Mutagenesis and Massively Parallel Sequencing
Source: PLoS Pathog. 2010 Oct 14;6(10):e1001146. doi: 10.1371/journal.ppat.1001146 (PMC2954836; doi:10.1371/journal.ppat.1001146)
Supplement: Figure S2 — Processing of vRNAs for massively parallel sequencing. A graphical representation of the protocol described in the text. Viral genomic RNAs (vRNAs) are isolated from the supernatant of infected cell cultures. RACE and RT-PCR are used to generate amplicons that span the entire viral genome. The amplicons are mixed in an equimolar ratio, and digested with AscI (added by the primer during PCR) to generate 5′ phosphorylated ends. This mix is then ligated into a random jumble of high molecular weight DNA (hmwDNA) to generate a starting material for sequencing library preparation. The hmwDNA is nebulized into fragments 400–800 bp in size, and the ends are polished to generate blunt, 5′ phosphorylated ends. Sequencing adapter “B” (shown in green) from the Roche library preparation kit is ligated onto both ends of the polished DNAs. This mixture is then digested with NotI to expose the ends of the transposon insertions, and a biotinylated modified adapter “A” (shown in blue) is ligated onto the exposed NotI ends. The mix is bound to streptavidin coated magnetic beads and washed to remove any fragments lacking the biotin tag. Templates for GS-FLX sequencing are eluted from the magnetic beads as single stranded DNA. (0.05 MB PDF) [file ppat.1001146.s002.pdf]

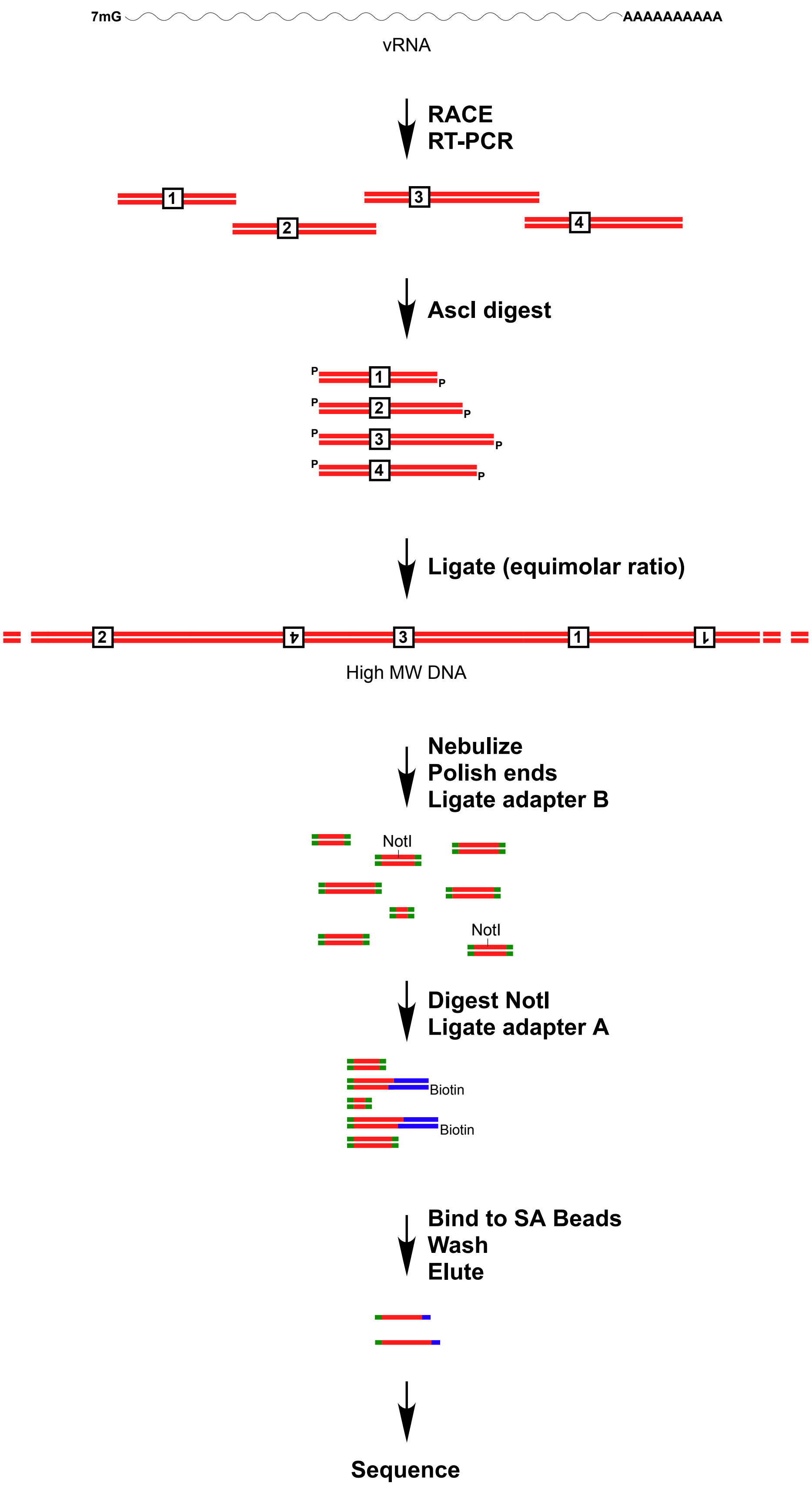
7mG
AAAAAAAAAA
vRNA
RACE
RT-PCR
1
2
3
4
AscI digest
P
Ligate (equimolar ratio)
High MW DNA
Nebulize
Polish ends
Ligate adapter B
NotI
Digest NotI
Ligate adapter A
Biotin
Bind to SA Beads
Wash
Elute
Sequence
