# Supplementary figures and images for: High-Resolution Functional Mapping of the Venezuelan Equine Encephalitis Virus Genome by Insertional Mutagenesis and Massively Parallel Sequencing
Source: PLoS Pathog. 2010 Oct 14;6(10):e1001146. doi: 10.1371/journal.ppat.1001146 (PMC2954836; doi:10.1371/journal.ppat.1001146)

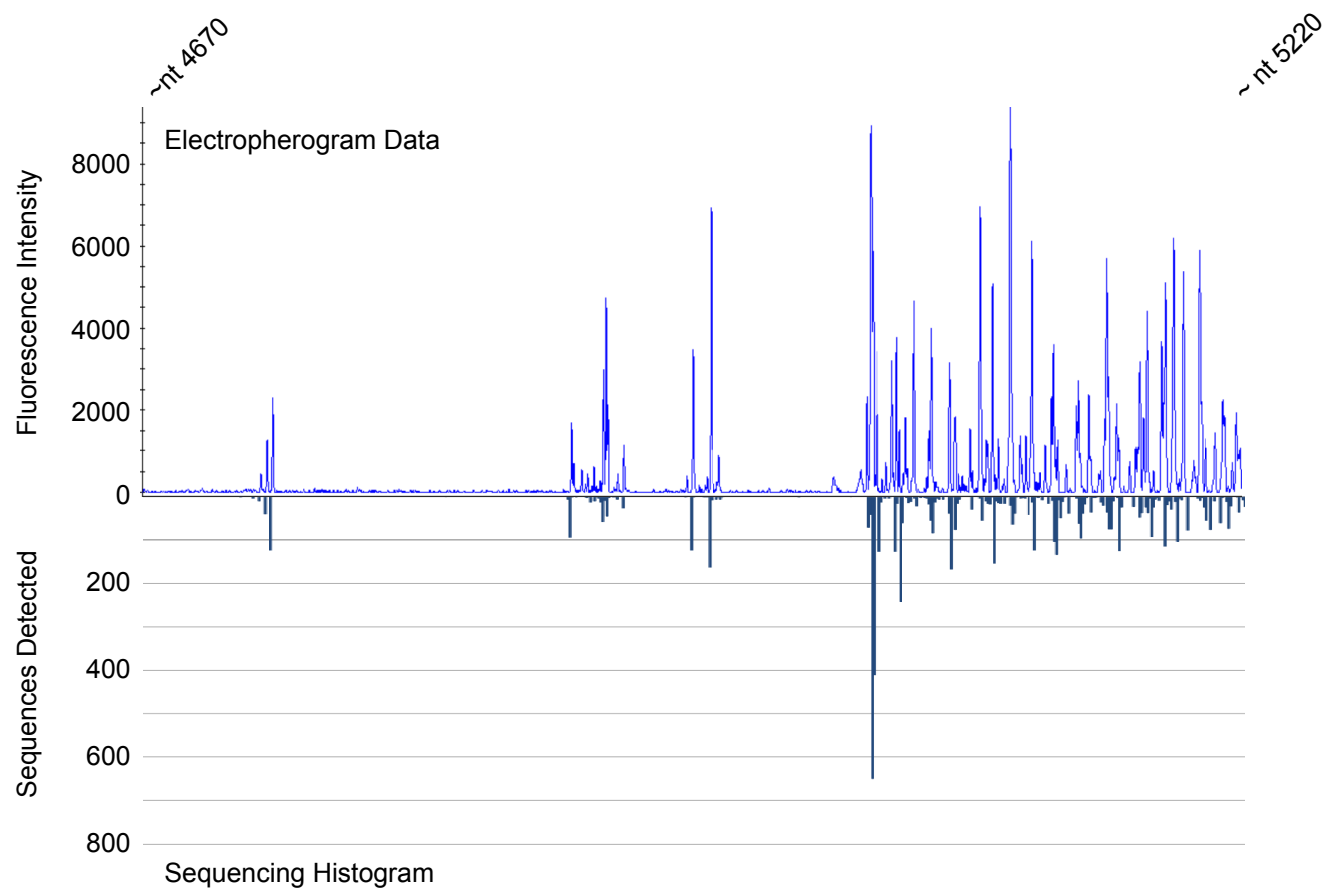

Supplement: Figure S3 — Comparison of electropherogram data to results obtained by sequencing. Electropherogram data (top), and sequencing histogram (bottom) from approximately nt 4670 - nt 5220 in nsP3, showing that similar results are obtained using both techniques. (0.10 MB PDF) [file ppat.1001146.s003.pdf]
