# Supplementary material for: High-Resolution Functional Mapping of the Venezuelan Equine Encephalitis Virus Genome by Insertional Mutagenesis and Massively Parallel Sequencing
Source: PLoS Pathog. 2010 Oct 14;6(10):e1001146. doi: 10.1371/journal.ppat.1001146 (PMC2954836; doi:10.1371/journal.ppat.1001146)
Supplement: Table S1 — Oligonucleotides used in this study. (0.03 MB PDF) [file ppat.1001146.s004.pdf]

Table S1: Oligonucleotides used in this study

BBU0002	FAM-CATTGGGTACGATCGCAAGGC
BBU0004	FAM-CAGTTGGCAGAGGCTTATGAGTCC
BBU0006	FAM-CTACAGCACAAGCGATGGCAAAAC
BBU0008	FAM-CTCCCAGCCTATATTGTTCTCAC
BBU0010	FAM-GGCCGCCCTCTGTATCTA
BBU0017	Biotin-ATATCCTTGGCCGCCTGGTGAAAC
BBU0018	Biotin-GATGGCTCCGGAGTCTCGTCTACC
BBU0019	Biotin-GTTGCCCCGCTGGTCACG
BBU0020	Biotin-GCACCCGCATCAAACCGTCAT
BBU0021	Biotin-ATCTCCAATTCGGTCCTCTC
BBU0245	CACGggcgcgccGACAGGGCCAGCGAAAGCATCA
BBU0246	CACGggcgcgccTTCGCCGAAAGCCGCCTCAAT
BBU0247	CACGggcgcgccGCTGATGGCGATGAATGAACACTG
BBU0248	CACGggcgcgccGTCAACCGCGTATACATCCTG
BBU0249	CACGggcgcgccGAGCTTCCCGCAGTTTGAGGTAG
BBU0250	CACGggcgcgccCCGGCTTCGTGGAGTCTGGA
BBU0253	CACGggcgcgccTGCCCGGGCCGAGACCACCTG
BBU0254	CACGggcgcgccGCGAGCACAGAATTAATACGACT
BBU0273	CACGggcgcgccCGCCGCGAGTTCTATGTA
BBU0274	CACGggcgcgccTCCGCGCACAAGAACACCGTCACT
BBU0277	CACGggcgcgccTCTTCCCTTGGCCTCCCCCTTTCT
BBU0278	CACGggcgcgccGTTCCCGTTCCAGCCAATGTATCC
V4450R	GCTACATCTGCATCAGTGGTG
V7394R	CTCTGAAAGAATACCCACTCG
V10603R	GGATTGTATATCTCCAA
3' RACE adapter	GCGAGCACAGAATTAATACGACTCACTATAGG$(T)_{12}$VN
BBU0323	GCCTTGCCAGCCCGCTCAG
BBU0324	CTGAGCGGGCTGGCAA
BBU0325	Biotin-GCCTCCCTCGCGCCATCAGAAAAC
BBU0326	GGCCGTTTTCTGATGGCGCGAGGGA
BBU0327	Biotin-GCCTCCCTCGCGCCATCAGGGGGC
BBU0328	GGCCGCCCCCTGATGGCGCGAGGGA

* AscI restriction sites indicated in lower case
