# Supplementary material for: High-Resolution Functional Mapping of the Venezuelan Equine Encephalitis Virus Genome by Insertional Mutagenesis and Massively Parallel Sequencing
Source: PLoS Pathog. 2010 Oct 14;6(10):e1001146. doi: 10.1371/journal.ppat.1001146 (PMC2954836; doi:10.1371/journal.ppat.1001146)
Supplement: Table S2 — Amplicons used in this study. (0.03 MB PDF) [file ppat.1001146.s005.pdf]

Table S2: Amplicons

PCR primers	Region Amplified
BBU0247+BBU0248	5' end to 515
BBU0249+BBU0250	104 to 4027
BBU0245+BBU0246	3825 to 6929
BBU0274+BBU0277	5450 to 7805
BBU0278+BBU0273	7564 to 11372
BBU0253+BBU0254	9823 to 3' end
BBU0002+BBU0017	3932 to 4684
BBU0004+BBU0018	4281 to 5059
BBU0006+BBU0019	4613 to 5350
BBU0008+BBU0020	4961 to 5701
BBU0010+BBU0021	5236 to 5803
