# Supplementary material for: High-Resolution Functional Mapping of the Venezuelan Equine Encephalitis Virus Genome by Insertional Mutagenesis and Massively Parallel Sequencing
Source: PLoS Pathog. 2010 Oct 14;6(10):e1001146. doi: 10.1371/journal.ppat.1001146 (PMC2954836; doi:10.1371/journal.ppat.1001146)
Supplement: Table S3 — Serum neutralizing titers for individual mice vaccinated with VEEV ts strains. 80% plaque reduction neutralization titers (PRNT80) of individual animals after inoculation with VEEV ts mutants. Titers registered as <20 indicate that no neutralization activity was seen at the lowest serum dilution (1∶20). Titers registered as >5120 indicate that plaque numbers were reduced >80% (compared to a no-serum control) at the highest dilution used in the assay (1∶5120). (0.03 MB PDF) [file ppat.1001146.s006.pdf]

Group	Mouse #	PRNT 80	Value Used for Calculations	Geometric Mean
**V3000, 10 PFU**	1	>5120	5120	226
	2	<20	10	
**ts3-1, 10 PFU**	1	4189	4189	2658
	2	>5120	5120	
	3	>5120	5120	
	4	4551	4551	
	5	>5120	5120	
	6	<20	10	
	7	>5120	5120	
	8	>5120	5120	
	9	>5120	5120	
	10	>5120	5120	
**ts3-1, 10000 PFU**	1	>5120	5120	4496
	2	>5120	5120	
	3	>5120	5120	
	4	>5120	5120	
	5	>5120	5120	
	6	>5120	5120	
	7	4189	4189	
	8	1920	1920	
	9	4551	4551	
	10	>5120	5120	
**ts3-3, 10 PFU**	1	>5120	5120	2441
	2	3650	3650	
	3	>5120	5120	
	4	3478	3478	
	5	4287	4287	
	6	>5120	5120	
	7	3922	3922	
	8	>5120	5120	
	9	>5120	5120	
	10	<20	10	
**ts3-3, 10000 PFU**	1	>5120	5120	3795
	2	>5120	5120	
	3	2633	2633	
	4	>5120	5120	
	5	3922	3922	
	6	3072	3072	
	7	4337	4337	
	8	3022	3022	
	9	>5120	5120	
	10	2168	2168	
**Double ts, 10 PFU**	1	2425	2425	3286
	2	>5120	5120	
	3	4851	4851	
	4	1920	1920	
	5	>5120	5120	
	6	1197	1197	
	7	>5120	5120	
	8	4551	4551	
	9	4287	4287	
	10	2071	2071	
**Double ts, 10000 PFU**	1	698	698	2887
	2	2973	2973	
	3	1982	1982	
	4	3922	3922	
	5	3686	3686	
	6	>5120	5120	
	7	>5120	5120	
	8	3178	3178	
	9	2025	2025	
	10	4007	4007	
**PBS**	1	<20	10	10
	2	<20	10	
	3	<20	10	
	4	<20	10	
	5	<20	10	
	6	<20	10	
	7	<20	10	
	8	<20	10	
	9	<20	10	
	10	<20	10	
